# Follicle-Stimulating Hormone Positively Associates with Metabolic Factors in Perimenopausal Women

**DOI:** 10.1155/2020/7024321

**Published:** 2020-11-12

**Authors:** Chen Zhang, Meng Zhao, Zhengyang Li, Yongfeng Song

**Affiliations:** ^1^Department of Endocrinology, Shandong Provincial Hospital Affiliated to Shandong First Medical University, Jinan 250021, Shandong, China; ^2^Shandong Institute of Endocrine & Metabolic Diseases, Jinan 250021, Shandong, China; ^3^Department of Endocrinology, Shandong Provincial Hospital Affiliated to Shandong University, Jinan 250021, Shandong, China

## Abstract

**Objective:**

Menopause is associated with the increased risk of metabolic syndrome (MetS) and cardiovascular (CV) disease. Most studies have focused the postmenopausal women and the relationships among estrogen, androgen, and Mets risk. The main aim of the study was to investigate the Mets risk in perimenopausal women and whether the variation of FSH is associated with metabolic factors.

**Methods:**

A single-center cross-sectional retrospective analysis including 154 premenopausal women and 124 perimenopausal women was performed.

**Results:**

The prevalence of Mets in the perimenopausal group was much higher than the premenopausal group (49.19% vs. 35.71%, *p* = 0.023). The prevalence of central obesity and NAFLD also increased in the perimenopausal group than in the premenopausal group. We grouped the population by FSH tertiles; compared with women in the lowest tertile, women in the highest tertile had higher age, WC, serum TC, LDL-C, AST, ALT, and creatine levels. The prevalence of hypertriglyceridemia, raised BP and Mets also increased in the highest tertile group. Further, we subdivided the perimenopausal women according to FSH tertiles. Compared with perimenopausal women in the lowest tertile, the prevalence of raised BP significantly increased in the highest tertile.

**Conclusions:**

The risk of Mets increased in perimenopausal females than in premenopausal women. And a higher FSH level was associated with higher WC, TG, BPs, and the risk of Mets in perimenopausal women. Elevated FSH level appears to be a risk factor of MetS biomarkers in perimenopausal women.

## 1. Introduction

Menopause, characterized by decreased serum estrogen level and increased follicle-stimulating hormone (FSH) level, is a normal part of a woman's life cycle, which has been associated with increased risk of metabolic syndrome (MetS), atherosclerosis, and cardiovascular (CV) disease [1, 2]. It was reported that the prevalence of MetS in general populations was 20–30% [3, 4] but increased up to 31–69% after menopause [5, 6].

Numerous studies have focused on the relationship between estrogen and metabolic factors, such as lipid, glucose, and obesity [7–9]. Besides the effect of estrogen, some studies paid attention to the increased FSH in menopause women. Sun et al. reported that FSH could stimulate the FSH receptor (FSHR) expressed on the surface of osteoclasts and directly regulated bone mass, resulting in osteoporosis, while estrogen could not correct this effect [10]. Our previous study found that serum FSH is positively correlated with total cholesterol (TC) levels and the prevalence of hypercholesterolemia in women, and blocking FSH inhibits hepatic cholesterol biosynthesis and reduces serum cholesterol [11].

Perimenopause is the transition period from the reproductive to the nonreproductive stage of life, with important changes in their hormone, including FSH and estrogen [12]. Women may experience a number of symptoms associated with the menopausal transition, including vasomotor symptoms, vaginal dryness, sleeping disorders, and the onset of depression [13], and menopause transition was also associated with adverse health indicators, such as increased TC and LDL-C levels, increased cardiovascular risk and a greater bone loss/increased bone turnover [14–16]. However, the MetS risks in perimenopause women and the risk factors remain unclear.

In this study, we performed a retrospective study to examine the risk of Mets and the association between FSH and components of MetS among Chinese perimenopausal women. To the best of our knowledge, the current analyses are a part of the few studies focusing on the relationship between FSH and metabolic factors in perimenopausal women.

## 2. Materials and Methods

### 2.1. Subjects

The cross-sectional study included females aged ≥40 years who participated in an epidemiological investigation performed in Ningyang County in the Shandong Province of China in 2014. All the participants had lived in their current residence for at least 5 years. All participants were instructed to complete a self-reported questionnaire, including detailed questions regarding the menopausal status, provided an overnight fasting blood sample, and underwent a medical examination. Written informed consent was obtained from each participant prior to data collection. This study was approved by the ethics committee of Shandong Provincial Hospital and the procedures were conducted in accordance with the Declaration of Helsinki.

The inclusion criterium was females aged ≥40 years with natural menstrual states. The exclusion criteria were as follows: (1) undergone hormone replacement therapy during the three months prior to the examination; (2) presence of complications or conditions that affect the menopausal status or lipid metabolism, such as ureterectomy, bilateral ovariectomy, premature ovarian failure, history of irregular menstruation during premenopause, pregnancy, lactation, psychiatric illness, acute cardiovascular or cerebrovascular disease, chronic respiratory disease, malignant tumors, or renal dysfunctions; (3) use of medications that affect the natural state of menopause or lipid metabolism, including estrogen, progesterone, androgen, glucocorticoids, statins, fibrates, thyroid hormones, antithyroid drugs or thiazide diuretics, over the preceding three months; (4) subjects with incomplete data.

As a result, 154 premenopausal and 124 perimenopausal female subjects were eligible for inclusion in our study.

### 2.2. Definition of Variables

The metabolic syndrome was defined according to the Adult Treatment Panel (ATP) III guidelines by the presence of three or more of the five following criteria [17, 18]: (1) waist circumference ≥80 cm for women; (2) serum triacylglycerol level ≥1.7 mmol/l; (3) HDL-cholesterol level <1.30 mmol/l for women; (4) fasting glucose level ≥5.6 mmol/l or use of glucose-lowering drug medication (insulin or oral agents); or (5) systolic blood pressure ≥130 mmHg and/or diastolic blood pressure ≥85 mmHg, and/or use of blood-pressure-lowering medication.

### 2.3. Data Collection

The determination of the menopausal status was based on the responses to questions regarding menstrual irregularity and amenorrhea in the self-reported questionnaire. Perimenopause was defined as the presence of menses within the past 3 months with a decrease in cycle predictability in the year preceding examination or 3–11 months of amenorrhea.

For the premenopausal participants, venipuncture was scheduled on days 2–5 of a spontaneous menstrual cycle to evaluate sex hormone levels. For the perimenopausal participants, fasting serum samples were randomly obtained [19]. Blood samples were drawn after 10 hours of overnight fasting. Biomedical analyses, including female sex hormones, serum lipid profiles, plasma glucose level, HbA1c level, serum uric acid (UA) level, and the parameters of hepatic and renal functions, were performed in the clinical laboratory center of Shandong Provincial Hospital.

Trained investigators obtained information on age, sex, and other essential data from the standardized questionnaire [20]. Height was determined to the nearest 1 cm, and weight was determined to the nearest 0.5 kg. Body mass index (BMI) was calculated as the weight in kilograms divided by the square of the height in meters. The waist circumference was measured in centimeters.

### 2.4. Statistical Analysis

All statistical analyses were performed using SPSS software (25.0 for Windows, SPSS Inc., Chicago, USA). The continuous variables data are presented as mean ± standard deviation, mean ± standard error, or median (interquartile range, if nonnormal distribution). Differences between the two groups were compared using the Student's *t*-test or the Mann–Whitney *U*-test (if nonnormal distribution). Differences among multiple groups were compared using one-way analysis of variance (followed by a multiple comparison test for subgroups using Least Significant Difference or Dunnett's T3 tests). All statistical tests were two-tailed, and statistical significance was defined as *p* < 0.05.

## 3. Results

### 3.1. Characteristics of the Study Population

The study population was divided into two groups according to the menstrual status: the perimenopausal group and the premenopausal group. The characteristics of the study population are shown in Table 1. The whole population contained 154 premenopausal and 124 perimenopausal female subjects. Compared with the premenopausal group, the perimenopausal females were older (50.06 ± 5.17 vs. 46.27 ± 2.50, *p* < 0.001), and the serum FSH and LH levels were elevated significantly while the serum estrogen levels were similar between the two groups. At the same time, the serum progesterone levels were decreased in perimenopausal females.

The body weight and BMI had no difference between the two groups, while the waist circumference was much higher in the perimenopausal group than the premenopausal group (90.10 ± 9.40 vs. 86.92 ± 9.22, *p* = 0.006). The serum TC, LDL-C, TG levels, and the liver enzyme (ALT and AST) and serum UA levels were much higher in perimenopausal females than in premenopause subjects. In addition, the blood pressure (SBP and DBP) levels, HDL-C levels, creatine levels, and thyroid function (FT3, FT4, TSH) had no significant difference between the two groups.

### 3.2. Risk of Mets Increased in Perimenopausal Females

As shown in Table 2, by definition, the overall prevalence of Mets in the whole study population was 41.73%. In the premenopausal group, 35.71% burdened from the Mets, while in the perimenopausal group, it was up to 49.19%, and the difference between the two groups was statistically significant (*p* = 0.023).

Further, we also observed the difference of different metabolic components between the two groups. Only the prevalence of central obesity increased in the perimenopausal group, while the prevalence of hypertriglyceridemia, raised FPG, hypertension, and low HDL had no difference between the two groups. Nonalcoholic fatty liver disease (NAFLD) often cooccurred with one or more MetS-associated phenotypes and they shared common pathogenic mechanisms [21]. We found that the prevalence of NAFLD also increased in the perimenopausal group than the premenopausal group (45.97% vs. 30.17%, *p* = 0.012).

### 3.3. FSH is Positively Associated with Risks of MetS Biomarkers

The serum FSH levels increased while the serum estrogen levels had no significant change in the perimenopausal group, so we grouped the population by FSH tertiles. The tertile ranges of FSH in the population were <8.55 (Q1), 8.55∼19.11 (Q2), >19.11 IU/L (Q3). As shown in Table 3, when we compared with women in the lowest tertile (Q1), women in the highest tertile (Q3) had higher age, WC, serum TC, LDL-C, AST, ALT, and creatine levels. The serum estrogen and progesterone levels were expected to decrease with FSH increased.

We also observed the prevalence of MetS biomarkers. Overall, compared with women in the lowest tertile (Q1), the prevalence of hypertriglyceridemia, raised BP, and Mets increased in the highest tertile (Q3) group. However, the prevalence of raised FPG and low HDL had no difference (Table 4).

Further, we subdivided the perimenopausal women into three groups according to FSH tertiles (Q1: <10.38, Q2: 10.38∼39.05, Q3: >39.05 IU/L) and examined the association of FSH with risks of MetS biomarkers in perimenopausal women. Compared with women in the lowest tertile (Q1), the prevalence of raised BP significantly increased in the highest tertile (Q3). The risk of Mets was higher in Q3 than in Q1, and the *p* value was close to the significance (*p* = 0.090). However, the prevalence of central obesity raised TG and FPG, and low HDL had no difference (Table 5). We noticed that the sample size was relatively small after classified into three groups (*n* = 40), and thus the statistic power might not be enough to detect a moderate effect.

## 4. Discussion

In this study, we found that the risk of Mets increased in perimenopausal females than in premenopausal women. A higher FSH level was associated with higher WC, TG, BPs, and the risk of Mets in perimenopausal women.

Midlife women have a high risk of Mets, which was reported that they had a one-in-three chance of developing MetS that predisposes them to T2DM and CVD [22, 23]. Midlife women usually underwent menopausal transition (MT) [24]. And the prevalence of the MetS increased from premenopause to postmenopause in women, independent of age [24]. Perimenopause is an intermediate stage between premenopause and postmenopause. This period corresponds to the final depletion of oocytes and can last up to 10 years, when women may experience important changes in their hormone [25]. However, The Mets risk in perimenopausal women is still unclear. To the best of our knowledge, this is one of the few studies to detect the association between FSH level and MetS biomarkers of normal perimenopausal women in a population-based investigation.

A hallmark of the menopausal transition is the dramatic reduction in estradiol levels, and with this reduction, there is a progressive shift toward androgen dominance in the hormonal milieu [26, 27]. Most of the previous studies have focused on the effects of estrogen and androgen fluctuation on Mets and CVD risk [28, 29]. Besides the reduction of estrogen, FSH levels increased sharply which was earlier than the estrogen reduction [25]. Strong correlations were found to exist between rising serum FSH levels and osteoporosis and obesity after menopause, and blocking FSH reduced body fat levels and increased the bone density in menopause [10, 30, 31]. Furthermore, postmenopausal women with a higher serum FSH (≥78.3 IU/L) had higher serum TC and LDL-C levels than those with relatively lower FSH levels (40–78.3 IU/L) [32], and FSH was positively associated with serum TC and LDL-C levels, which was independent of age and estrogen [11]. It was reported that even with regular menstruation, women with FSH ≥7 IU/L had higher TC and LDL levels than those with FSH <7 IU/L [33]. All these results suggested that FSH was also of great importance in metabolic regulation. Likewise, FSH receptor polymorphism seemed to play an important role in increased Mets risk in women. Cannarella R et al. have demonstrated that FSHR gene polymorphism, rs6166, significantly influences glucose metabolism [34]. Moreover, it was reported that women having activated FSHR polymorphisms also have lower bone mass and higher bone turnover [35]. In this study, we also found that a higher FSH level was associated with higher WC, TG, BPs, and the risk of Mets in perimenopausal women.

Central obesity was the most frequent component of Mets for midlife women [2]. In our study, we also found that there was no difference in weight and BMI between premenopausal and perimenopausal women, while the waist circumference increased in perimenopausal women, and the prevalence of central obesity was highest than other Mets components. The WC levels increased with the FSH level increase. These associations might be explained by the involvement of FSH in fat accumulation and redistribution in humans. FSH was found to bind to FSHR in adipocytes and promote excessive lipid biosynthesis and lipid droplet formation in visceral adipose tissue through the G*α*i/Ca^2+^/CREB pathway [36]. Moreover, blocking FSH activated thermogenic adipose tissue and reduced adiposity [31]. A previous study also found that (log)FSH change was positively correlated with (log)fat mass change after menopause [37]. These results suggested that central obesity might be the pathological basis for metabolic disturbance in perimenopausal women.

Some studies also reported the associations between FSH and other Mets components in postmenopausal women. SBP and DBP increased by 6.11 and 3.54 mmHg after menopause, respectively [38]. One study concluded that the increase in FSH was related to an increase in blood pressure in postmenopausal women [39]. In that study, the intracellular mechanism of FSH promoting catecholamine synthesis in PC12 and rat adrenal medulla chromaffin cells was proposed. The SPECT-China study found that low FSH was associated with prediabetes and diabetes in postmenopausal women [40]. Sun et al. also reported that postmenopausal women in the lower FSH level had higher FBG [41]. However, in our study, we did not find any difference for FPG and blood pressure in premenopausal and perimenopausal women. While FSH increased, the prevalence of raised BP also increased. The inconsistent results may be due to the different study population. We used perimenopausal women while other studies used postmenopausal subjects. In contrast, the metabolic disturbance was much more serious in postmenopausal subjects than perimenopausal women.

Our study also has some limitations. The main limitation was the small samples. We only enrolled 154 premenopausal women and 124 perimenopausal women. Some phenomenon may be masked by the small samples. Second, this was a retrospective study that cannot establish causal relationships. A large-scale, randomized, prospective study would be a better study design to investigate the relationship between FSH and MetS, as well as MetS biomarkers. Third, we used a self-reported questionnaire to determine menopausal status but did not have the Anti-Müllerian hormone (AMH), which is a reliable indicator of ovarian reserve and menopausal status [42]. Fourth, the lack of insulin levels and HOMAi limited our further explorations of their associations with FSH levels.

In conclusion, the risk of Mets increased in perimenopausal females. A higher FSH level appears to be a risk factor of MetS biomarkers in perimenopausal women. However, further clinical and mechanistic studies are needed to explore the effect of FSH on metabolism disturbance.

## Figures and Tables

**Table 1:** The characteristics of the study population.

	Premenopause	Perimenopause	*p* value
	*N* = 154	*N* = 124	
Age (ys)	46.27 ± 2.50	50.06 ± 5.17	<0.001
Weight (kg)	65.92 ± 10.20	66.41 ± 10.30	0.697
WC (cm)	86.92 ± 9.22	90.10 ± 9.40	0.006
BMI (kg/m^2^)	25.86 ± 3.52	26.60 ± 3.77	0.095
SBP (mmHg)	130.49 ± 20.31	132.00 ± 20.78	0.544
DBP (mmHg)	78.56 ± 12.90	78.71 ± 10.86	0.919
FT3 (pmol/L)	4.71 ± 0.48	4.88 ± 1.15	0.114
FT4 (pmol/L)	15.49 ± 2.02	15.42 ± 3.50	0.842
TSH (mIU/L)	2.95 ± 1.67	2.72 ± 1.65	0.270
FSH (IU/L)	9.30 (6.45∼13.93)	20.87 (8.06∼54.87)	<0.001
LH (IU/L)	6.00 (4.10∼8.47)	16.64 (6.60∼32.32)	<0.001
E2 (pg/ml)	55.12(31.92∼98.29)	61.11(19.16∼146.80)	0.851
PRG (ng/ml)	0.52 (0.33∼1.74)	0.39 (0.20∼0.97)	0.003
FPG (mmol/L)	5.78 ± 1.49	5.81 ± 1.51	0.906
HbA1c (%)	5.75 ± 0.94	5.81 ± 0.79	0.604
TC (mmol/L)	4.78 ± 0.85	5.20 ± 0.95	<0.001
LDL-C (mmol/L)	2.73 (2.32∼3.08)	2.97 (2.48∼3.45)	0.001
HDL-C (mmol/L)	1.39 (1.20∼1.55)	1.36 (1.18∼1.62)	0.900
TG (mmol/L)	0.95 (0.78∼1.34)	1.07 (0.83∼1.71)	0.023
ALT (U/L)	13.85 ± 5.42	17.02 ± 6.56	<0.001
AST (U/L)	17.57 ± 4.86	19.77 ± 4.85	<0.001
Crea (*μ*mol/L)	61.79 ± 6.16	62.71 ± 5.36	0.096
UA (*μ*mol/L)	263.42 ± 59.82	291.12 ± 72.90	0.001

All data are expressed as mean ± standard deviation or median with interquartile range. Abbreviations: WC: waist circumference; BMI: body mass index; SBP: systolic blood pressure; DBP: diastolic blood pressure; FT3: free triiodothyronine; FT4: free thyroxine; TSH: thyroid stimulating hormone; FSH: follicle stimulating hormone; LH: luteinizing hormone; E2: estrogen; PRG: progesterone; FPG: fasting plasma glucose; TC: total cholesterol; LDL-C: low density lipoprotein cholesterol; HDL-C: high density lipoprotein cholesterol; TG: triglyceride; ALT: Alanine Aminotransferase; AST: Aspartate transaminase; Crea: creatine; UA: uric acid.

**Table 2:** The risk of Mets increased in perimenopausal females.

Components of MetS, *n*(%)	Premenopause *n* = 154	Peri-menopause *n* = 124	Total *n* = 278	*p*
Central obesity	116(77.33)	106(88.33)	222(82.22)	0.019
Raised TG	25(16.23)	31(25.00)	56(20.14)	0.070
Low HDL-C	62(40.26)	55(44.35)	117(42.09)	0.492
Raised FPG	65(42.21)	57(45.97)	122(43.88)	0.530
Raised BP	69(46.00)	67(54.03)	136(49.64)	0.186
MetS	55(35.71)	61(49.19)	116(41.73)	0.023
NAFLD	35(30.17)	57(45.97)	92(38.33)	0.012

Data are expressed as number with proportion for categorical variables. Pearson's chi-squared test was used in the comparison of dichotomous variables.

**Table 3:** The characteristics of the study population with different FSH levels.

	Serum FSH tertiles			*p* value
	Q1	Q2	Q3	
Age (ys)	47.82 ± 5.18	46.65 ± 2.78	50.13 ± 4.62*∗*	<0.001
Weight (kg)	66.39 ± 10.09	64.43 ± 10.03	67.33 ± 10.48	0.199
WC (cm)	88.57 ± 8.74	86.12 ± 9.39	90.73 ± 8.92$$	0.008
BMI (kg/m^2^)	26.19 ± 3.54	25.52 ± 3.75	26.74 ± 3.56$	0.110
SBP (mmHg)	132.09 ± 23.02	127.87 ± 18.01	134.63 ± 21.80$	0.128
DBP(mmHg)	78.11 ± 13.63	78.15 ± 11.68	79.17 ± 11.06	0.826
E2 (pg/ml)	120.40(63.20, 161.70)	60.11(34.18, 95.86)*∗*	22.16(13.08, 46.36)*∗∗*$	<0.001
PRG (ng/ml)	0.95(0.39, 9.46)	0.54(0.32, 1.56)*∗*	0.30 (0.16, 0.47)*∗∗*$	<0.001
FPG (mmol/L)	5.59 ± 1.01	5.79 ± 1.49	5.96 ± 1.80	0.282
HbA1c (%)	5.65 ± 0.54	5.76 ± 0.93	5.91 ± 1.06	0.171
TC (mmol/L)	4.82 ± 0.82	4.89 ± 0.91	5.29 ± 1.04*∗∗*$$	0.003
LDL-C (mmol/L)	2.76 ± 0.69	2.79 ± 0.61	3.07 ± 0.80*∗∗*$	0.014
HDL-C (mmol/L)	1.38 ± 0.26	1.40 ± 0.23	1.41 ± 0.34	0.771
TG (mmol/L)	0.97(0.72, 1.38)	0.95(0.77, 1.33)	1.09(0.84, 1.77)	0.597
ALT (U/L)	14.57 ± 5.70	14.49 ± 6.39	16.99 ± 6.23*∗*$	0.016
AST(U/L)	18.23 ± 4.05	17.82 ± 4.94	20.10 ± 5.04*∗*$$	0.006
Crea (*μ*mol/L)	60.46 ± 5.56	62.24 ± 4.76	63.82 ± 6.73*∗*	0.001
UA (*μ*mol/L)	278.21 ± 60.35	271.37 ± 77.60	290.54 ± 65.67	0.245

All data are expressed as mean ± standard deviation or median with interquartile range. The tertile ranges of FSH in the population were <8.55 (Q1), 8.55∼19.11 (Q2), >19.11 IU/L (Q3). *∗p* < 0.05, *∗p* < 0.01 vs Q1 group; $*p* < 0.05, $$*p* < 0.01 vs. Q2 gro.

**Table 4:** FSH is positively associated with risks of MetS biomarkers.

Components of MetS, *n*(%)	Q1 *n* = 79	Q2 *n* = 79	Q3 *n* = 78	*p* value
Central obesity	66(86.84)	57(73.08)^*∗*^	68(90.67)^$$^	0.009
Raised TG	14(17.72)	9(11.39)	21(26.92)^$^	0.043
Low HDL-C	31(39.24)	31(39.24)	34(43.59)	0.815
Raised FPG	32(40.51)	34(43.04)	38(48.72)	0.570
Raised BP	33(41.77)	36(45.57)	49(62.82)^*∗∗*$^	0.019
Mets	27(34.17)	26(32.91)	38(48.72)^*∗*^	0.043

Data are expressed as number with proportion for categorical variables. Pearson chi-squared test was used in the comparison for dichotomous variables. *∗p* < 0.05, *∗p* < 0.01 vs Q1 group; $*p* < 0.05, $$*p* < 0.01 vs. Q2 group.

**Table 5:** The risks of MetS biomarkers according to FSH tertiles in perimenopausal women.

Components of MetS, *n*(%)	Q1 *n* = 40	Q2 *n* = 41	Q3 *n* = 40	*p* value
Central obesity	34(87.18)	32(84.21)	38(92.50)	0.490
Raised TG	8(20.00)	9(21.95)	12(30.00)	0.539
Low HDL-C	20(50.00)	15(36.59)	18(45.00)	0.469
Raised FPG	13(32.50)	21(51.22)	21(52.50)	0.131
Raised BP	18(45.00)	19(46.34)	29(72.50)^∗$^	0.020
Mets	17(43.69)	18(43.90)	26(65.00)	0.090

Data are expressed as number with proportion for categorical variables. Pearson chi-squared test was used in the comparison for dichotomous variables. *∗p* < 0.05, *∗p* < 0.01 vs Q1 group; $*p* < 0.05, $$*p* < 0.01 vs. Q2 group.

## Data Availability

The data sets used and analyzed during the current study could be made available upon reasonable request to the corresponding author.
